# Thyroid Function in Adults with Prader–Willi Syndrome; a Cohort Study and Literature Review

**DOI:** 10.3390/jcm10173804

**Published:** 2021-08-25

**Authors:** Karlijn Pellikaan, Fleur Snijders, Anna G. W. Rosenberg, Kirsten Davidse, Sjoerd A. A. van den Berg, W. Edward Visser, Aart J. van der Lely, Laura C. G. de Graaff

**Affiliations:** 1 Department of Internal Medicine, Division of Endocrinology, Erasmus Medical Center, University Medical Centre Rotterdam, 3015 GD Rotterdam, The Netherlands; k.pellikaan@erasmusmc.nl (K.P.); f.snijders@erasmusmc.nl (F.S.); a.rosenberg@erasmusmc.nl (A.G.W.R.); k.davidse@erasmusmc.nl (K.D.); s.a.a.vandenberg@erasmusmc.nl (S.A.A.v.d.B.); w.e.visser@erasmusmc.nl (W.E.V.); a.vanderlelij@erasmusmc.nl (A.J.v.d.L.); 2 Department of Internal Medicine, Division of Endocrinology, Center for Adults with Rare Genetic Syndromes, Erasmus Medical Center, University Medical Center Rotterdam, 3015 GD Rotterdam, The Netherlands; 3 Dutch Center of Reference for Prader–Willi Syndrome, 3015 GD Rotterdam, The Netherlands; 4 Academic Centre for Growth Disorders, Erasmus Medical Center, University Medical Centre Rotterdam, 3015 GD Rotterdam, The Netherlands; 5 Department of Clinical Chemistry, Erasmus Medical Center, University Medical Centre Rotterdam, 3015 GD Rotterdam, The Netherlands; 6 Department of Internal Medicine, Academic Center for Thyroid Diseases, Erasmus Medical Center, 3015 GD Rotterdam, The Netherlands

**Keywords:** Prader–Willi syndrome, thyroid hormones, hypothyroidism

## Abstract

Prader–Willi syndrome (PWS) is a complex genetic syndrome combining hypotonia, hyperphagia, a PWS-specific neurocognitive phenotype, and pituitary hormone deficiencies, including hypothyroidism. The low muscle mass associated with PWS causes a low energy expenditure due to a low basal metabolic rate. Combined with increased energy intake due to hyperphagia, this results in a high risk of obesity and associated cardiovascular disease. To reduce the high mortality in PWS (3% yearly), exercise is extremely important. As hypothyroidism can impair exercise tolerance, early detection is crucial. We performed a literature search for articles on hypothyroidism in PWS, measured thyroid hormone (TH) levels in 122 adults with PWS, and performed a medical file search for medication use. Hypothyroidism (low free thyroxin) was present in 17%, and often central in origin (80%). Triiodothyronine levels were lower in patients who used psychotropic drugs, while other TH levels were similar. One in six patients in our cohort of adults with PWS had hypothyroidism, which is more than in non-PWS adults (3%). We recommend yearly screening of free thyroxin and thyroid-stimulating hormone levels to avoid the negative effects of untreated hypothyroidism on basal metabolic rate, body mass index, and cardiovascular risk. Additionally, we recommend measuring TH concentrations 3–4 months after the start of growth hormone treatment.

## 1. Introduction

Prader–Willi syndrome (PWS) is a rare, complex, multisystem condition with an estimated prevalence of 1/10,000 to 1/30,000 [1]. It is caused by loss of expression of a cluster of maternally imprinted genes on chromosome 15q11.2-q13, most commonly caused by a paternal deletion (65–75%) or a maternal uniparental disomy (mUPD, 20–30%). In rare cases, PWS is caused by imprinting center defects (ICD, 1–3%) or paternal chromosomal translocations (0.1%) [2,3]. Features of PWS include hyperphagia, hypotonia, and delayed psychomotor development. In addition, hypothalamic dysfunction could result in abnormal temperature regulation, disturbed pain registration, and pituitary hormone deficiencies, including hypothyroidism. There are contradictory data regarding the prevalence of hypothyroidism in PWS [4,5].

Due to hypotonia and the low muscle mass associated with the syndrome, adults with PWS have a low basal metabolic rate (BMR) which, combined with hyperphagia, increases the risk of developing obesity. To increase energy expenditure and compensate for this low BMR, exercise is extremely important. However, if left untreated, hypothyroidism can cause fatigue and exercise intolerance [6,7,8,9]. This leads to a further decrease in muscle mass and BMR, an increase in body mass index (BMI), and increased cardiovascular risk [10,11,12]. As mortality in PWS is high (3% yearly in children and adults, and 7% yearly in adults with PWS above 30 years old) and often related to obesity and cardiovascular problems (e.g., cardiac failure and pulmonary embolism), it is of utmost importance to treat hypothyroidism and other factors affecting BMR at an early stage [6,7,8,9,13,14,15].

Apart from affecting BMR, hypothyroidism can also have more direct cardiovascular effects. Low thyroid hormone (TH) levels have been associated with diastolic hypertension, increased systemic vascular resistance leading to decreased cardiac output, myocardial stiffness, left ventricular diastolic dysfunction, accelerated atherosclerosis, and coronary artery disease [16]. Even subclinical hypothyroidism has been associated with an increased risk of coronary heart disease and mortality [17]. Treatment with levothyroxine reduces low-density lipoprotein cholesterol, total cholesterol, hypertension, and diastolic dysfunction, and delays atherosclerosis [16].

Our clinical experience is that hypothyroidism is frequently missed in adults with PWS. The PWS-specific behavioral phenotype, physicians’ unawareness of the PWS-specific diagnostic pitfalls, and the lack of medical guidelines for adults with PWS can cause both patients’ and doctors’ delay. The intellectual disability present in most PWS adults makes it difficult for patients to express symptoms such as fatigue and constipation. In addition, physicians often falsely assume that fatigue or excessive daytime sleepiness are inherent to the syndrome, and do not perform further investigations. Moreover, reduced appetite and weight gain—other important symptoms of hypothyroidism—are hard to recognize, as adults with PWS have hyperphagia and are often on a diet. Therefore, hypothyroidism can easily be missed if not actively screened for.

In 122 adults with PWS, we performed TH measurements and reviewed medical files for clinical data, including use of medication. As few large studies have investigated TH measurements in adults with PWS, we provide a thorough exploratory analysis of the patient characteristics possibly associated with TH concentrations (gender, genotype, BMI, age, growth hormone (GH) treatment, and use of psychotropic drugs). Additionally, we searched the literature for the prevalence and mechanisms of hypothyroidism (central or primary hypothyroidism) in adults with PWS. Based on our findings, we present recommendations for the screening and management of hypothyroidism in adults with PWS.

## 2. Materials and Methods

Ethical review and approval were waived for this study by the Medical Ethics Committee of the Erasmus University Medical Center, Rotterdam, the Netherlands. In this retrospective study, we reviewed the medical files of adults that visited the multidisciplinary outpatient clinic of our PWS reference center in the Erasmus University Medical Center, between January 2015 and December 2020, and underwent our routine systematic health screening. This systematic screening consists of a structured interview, a complete physical examination, a medical questionnaire, a review of the medical file including medication use, biochemical measurements and, if indicated and feasible, additional tests, as described previously (see [18]).

During the visit, blood samples were taken for general medical screening, including evaluation of thyroid function (fT4, triiodothyronine (T3), TSH). The reference values in our center for TSH were 0.4–4.3 mU/L before 1 February 2019, and 0.56–4.27 mU/L after that date. The reference values for fT4 were 11–25 pmol/L (Ortho Vitros^®^ assay, Vitros ECI Immunodiagnostic System; Ortho-Clinical Diagnostics, Rochester, MI, USA) before 12 April 2019, and 13.5–24.3 pmol/L after that date (Fuijrebio Lumipulse^®^ assay). Reference values in our center for T3 were 1.4–2.5 nmol/L before 12 April 2019, and 0.7–2.0 nmol/L after that date. TSH, fT4 and T3 measurements changed methods during the study, but they were calibrated similarly, as checked by external quality assessment schemes.

Overt hypothyroidism was defined as an fT4 concentration below the reference range. Central overt hypothyroidism was defined as an fT4 concentration below the reference range, with a TSH concentration below or within the reference range. Primary hypothyroidism was defined as an fT4 concentration below the reference range, with a TSH concentration above the reference range. Overt hyperthyroidism was defined as an fT4 concentration above the reference range. If patients used levothyroxine before visiting our reference center, the diagnosis of overt hypothyroidism was based on referral letters and/or laboratory measurements before the start of levothyroxine; in that case, the distinction between primary or central hypothyroidism was also based on referral letters or, if available, on the laboratory measurements before the start of levothyroxine compared to the local reference values.

Subclinical hypothyroidism was defined as a normal fT4 concentration, with a TSH concentration above the reference range, based on a single measurement. It is important to note that the diagnosis of subclinical hypothyroidism is less reliable in adults with PWS. In the general population, TSH can be affected by obesity [19,20,21,22]. Furthermore, hypothyroidism can be both primary and central in PWS. Taken together, this means that TSH and, therefore, the diagnosis of subclinical hypothyroidism, should be interpreted with caution.

We investigated the relationship between TH measurements and genotype, as this relationship is still largely unknown. As gender, age, BMI, and GH treatment are known to influence TH in the general population, we also investigated their effect on TH concentrations in our cohort of adults with PWS [23,24,25,26,27,28,29,30].

### 2.1. Literature Review

We performed a search on Embase, Medline, the Web of Science Core Collection, the Cochrane Central Register of Controlled Trials, and Google Scholar for articles that describe thyroid function and/or TH measurements in patients with PWS. The search was last updated on 22 July 2021. For the full search strategy, see Table S1.

Inclusion criteria were: original research articles that described the prevalence of thyroid abnormalities (including, but not limited to: central and primary hypothyroidism, hyperthyroidism, and subclinical hypothyroidism) or TH measurements (including, but not limited to: thyroxine (T4), fT4, T3, free T3 (fT3), reverse T3 (rT3), and TSH) in methylation-positive individuals with PWS. Exclusion criteria were: meeting reports, workshop summaries, conference abstracts, guidelines, articles that included 10 or fewer subjects with PWS, articles that were not available online, and articles that were not available in English. When the same population was described in multiple articles, the population with the most laboratory values or the largest population was included. When an article described thyroid function before and after the start of GH treatment, only data at baseline were included in the table. Authors were contacted to clarify data when needed.

### 2.2. Statistical Analysis

Statistical analysis was performed using R version 3.6.3. Descriptive statistics for continuous variables are reported as median (interquartile range (IQR)). For dichotomous variables we display the number of patients and the percentage of the total number of patients, *n* (%). We used a chi-squared test to compare the prevalence of hypothyroidism between males and females, between paternal deletion and mUPD, between patients who did and did not use GH treatment, and between patients who did and did not use psychotropic drugs. To investigate the relationship between hypothyroidism, and BMI and age, we used the Wilcoxon rank sum test. We also used the Wilcoxon rank sum test to investigate the relationships between gender, genotype, and use of GH treatment and psychotropic drugs on the one hand, and laboratory measurements (fT4, T3, and TSH) on the other hand. If there were ties, an exact calculation method was used. The Kendall rank correlation test was used to assess correlations between age and BMI on the one hand, and laboratory measurements (fT4, T3, and TSH) on the other hand. As this was an exploratory analysis, no correction for multiple testing was performed.

## 3. Results

### 3.1. Baseline

Baseline characteristics of the 122 adults with PWS participating in the study are shown in Table 1. The median age was 29 years (IQR 21–39), and the median BMI was 29 kg/m^2^ (IQR 26–36). We included 58 males and 64 females. Paternal deletion was the most common genotype (*n* = 66, 54%), followed by mUPD (*n* = 43, 35%). A total of 63 patients (52%) had never received GH treatment, while 43 patients (35%) received GH treatment at the time of the study. Medication use before our systematic screening included use of hydrocortisone (4 adults daily and 49 adults only during physical or psychological stress), estrogen replacement therapy (34/64 females), testosterone replacement therapy (24/58 males), and thyroid hormone replacement therapy (*n* = 19, 16%). A total of 67 (55%) patients lived in a non-specialized facility, 24 (20%) in a specialized PWS group home, and 31 (25%) with family. Most patients had received special education (*n* = 87, 71%).

### 3.2. Hypo- and Hyperthyroidism

Hypothyroidism was present in 21 patients (17%). A total of 12 patients had central hypothyroidism, 3 patients had primary hypothyroidism, and in 6 patients it was unknown whether the hypothyroidism was central or primary. In 17 patients, the diagnosis of hypothyroidism was based on referral letters. TH concentrations were provided in the referral letter in five cases. Additionally, two patients were diagnosed during childhood by the pediatric endocrinology department at our reference center, and two patients were diagnosed during our systematic health screening. The median age at diagnosis of hypothyroidism was 18 years (IQR 13–27) (age at diagnosis was unknown in two patients). The median dose of levothyroxine in patients with hypothyroidism was 68.8 μg (IQR 50.0–100.0) daily. Additionally, one patient with very mild hypothyroidism did not receive any treatment.

Three patients (2%) had a normal fT4 concentration, with a TSH concentration above the reference range (subclinical hypothyroidism), while one patient was diagnosed with hyperthyroidism, treated with thiamazole. Although not statistically significant, hypothyroidism seemed to be more prevalent in females (23%) than in males (10%, *p* = 0.051). There was no relationship with genotype, age, BMI, or GH treatment (Table 2, Table 3 and Table 4).

### 3.3. Thyroid Hormone Levels

**Figure 1:**
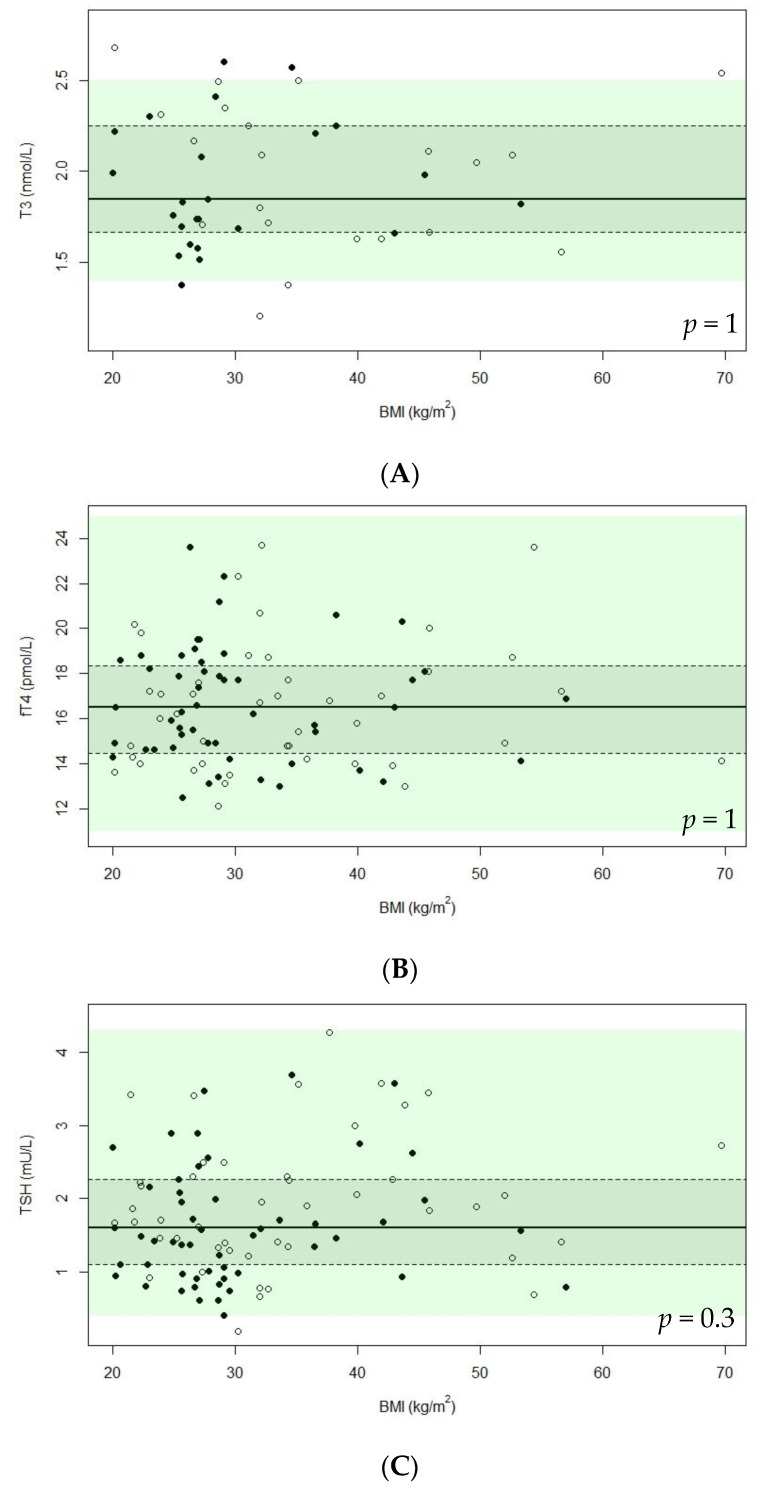
Scatterplots of T3, fT4, and TSH in relation to BMI. Abbreviations: body mass index (BMI), free thyroxine (fT4), triiodothyronine (T3), thyroid-stimulating hormone (TSH). Only patients without hyperthyroidism, hypothyroidism, or subclinical hypothyroidism are depicted in this figure. Legends: males are depicted by closed dots and females by open dots. The solid line represents the median, while the dashed line with the grey rectangle represents the interquartile range. The reference range is given as a green, transparent rectangle. (**A**) T3 vs. BMI for males and females; (**B**) fT4 vs. BMI for males and females; (**C**) TSH vs. BMI for males and females. Reference values: TSH: before 1 February 2019: 0.4–4.3 mU/L (*n* = 69), after 1 February 2019: 0.56–4.27 mU/L (*n* = 28); fT4: before 12 April 2019: 11–25 pmol/L (*n* = 73), after 12 April 2019: 13.5–24.3 pmol/L (*n* = 22); T3: before 12 April 2019: 1.4–2.5 (*n* = 33), after 12 April 2019: 0.7–2.0 nmol/L (*n* = 12). Only the reference range that was valid for the most observations is shown.

For the 97 patients without (subclinical) hypo- or hyperthyroidism, TH concentrations and the associations between TH concentrations and patient characteristics (gender, genotype, age, BMI, and use of GH treatment and psychotropic drugs) are shown in Table 2, Table 3 and Table 4. The median fT4 concentration was 16.5 pmol/L (IQR 14.3–18.5), the median T3 concentration was 1.9 nmol/L (IQR 1.7–2.3), and the median TSH concentration was 1.6 mU/L (IQR 1.1–2.3). T3 was significantly lower in older patients and in patients without current GH treatment, while fT4 and TSH levels were similar. Gender, genotype, and BMI were not significantly related to any of the TH measurements. To visualize the exact distribution of the TH concentrations, we show the T3, fT4, and TSH concentrations according to BMI in Figure 1A–C.

### 3.4. Psychotropic Drugs

We explored the relationship between TH concentrations and the use of psychotropic or antiepileptic drugs. Forty-nine patients used psychotropic drugs. Only two patients used antiepileptic medication, and both also used psychotropic drugs. Therefore, the relationship between the use of antiepileptic drugs and thyroid function was not further explored. Use of psychotropic drugs was not associated with hypothyroidism (Table 4). However, T3 was significantly lower in patients who used psychotropic drugs (median 1.7 nmol/L (IQR 1.6–2.0)) than in those who did not (median 2.1 nmol/L (IQR 1.7–2.3), *p* = 0.02). The mean age of patients using psychotropic drugs was 36 years, and the mean age of patients not using psychotropic drugs was 29 years. No associations for specific types of psychotropic drugs were found (Table S2 and S3).

### 3.5. Literature Review

The results of our literature review are summarized in Table 5, Table 6 and Table 7. Only 5 studies reported thyroid function separately for adults, while the other 21 studies reported thyroid function in children (*n* = 12) or in mixed populations containing children and adults (*n* = 9). Paternal deletion was the most common genotype in all studies. The prevalence of hypothyroidism differed between 0% and 33% in most studies, with one study reporting a prevalence of 72%. However, this study only included 18 children with PWS who were only up to 2 years old and, in this study, thyroid axis dysfunction was defined as serum total T4 and/or serum fT4 levels below the 2.5th percentile of a reference population. The prevalence of hypothyroidism in studies that only included adults ranged between 5% and 13%. For mixed populations of both children and adults, this prevalence was between 0% and 26%. Only two studies that included adults reported whether the hypothyroidism was central or primary in origin [31,32]. Although central hypothyroidism was more prevalent (2% and 4%), primary hypothyroidism (0% and 2%) did also occur. One study reported on the prevalence of subclinical hypothyroidism in PWS, and showed a prevalence of 5% in children and 1% in adults. Additionally, 11 studies reported TSH, 5 total T4, 14 fT4, 5 total T3, 6 free T3, and 1 reverse T3 concentrations.

### 3.6. Clinical Recommendations

**Figure 2:**
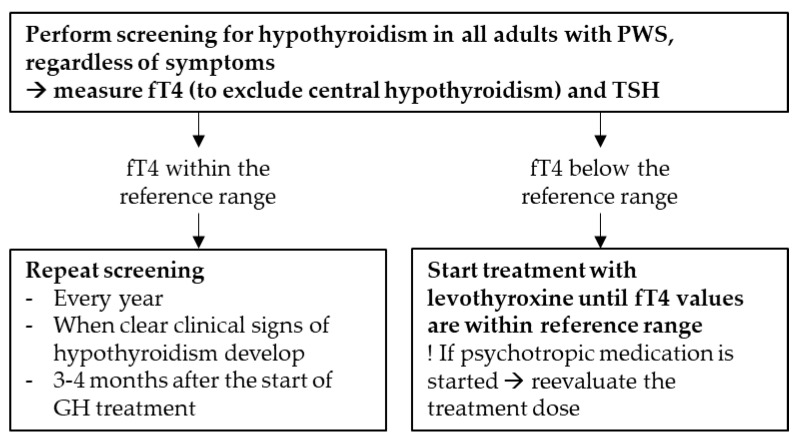
Recommendations for the screening and treatment of hypothyroidism in adults with Prader–Willi syndrome.

Based on the results of our cohort, the literature review, and our clinical expertise, we formulated practical clinical recommendations for the screening and treatment of hypothyroidism in adults with PWS (Figure 2).

## 4. Discussion

The prevalence of hypothyroidism detected in our cohort of 122 adults with PWS was 17%, compared to only 3% in non-PWS adults [56]. The risk of hypothyroidism was increased in all adults with PWS, regardless of gender, genotype, age, BMI, or use of GH treatment or psychotropic drugs.

Our prevalence of hypothyroidism was higher than that of most previous studies on hypothyroidism in adults and mixed cohorts of adults and children (Table 5 and Table 6). However, two large French studies both showed an even higher prevalence of hypothyroidism (26%) in patients with PWS of 16 years and older [47,48]. This indicates that, although the prevalence is variable, hypothyroidism is frequent in adults with PWS.

Compared to the general population, there are several aspects of PWS that increase the complexity of the diagnosis and treatment of hypothyroidism in these patients. An increased vulnerability to the effects of untreated hypothyroidism of the patients, diagnostic challenges, and altered TH metabolism make hypothyroidism a complex issue in adults with PWS.

### 4.1. Vulnerability of the Patients

The vulnerability of the patients makes the treatment of hypothyroidism an important topic. The common effects of hypothyroidism on the muscles and the brain can be especially harmful to adults with PWS, as they already have impaired exercise tolerance and brain function.

#### 4.1.1. Exercise Intolerance and Cardiovascular Risk

Patients with PWS have a high risk of developing obesity due to hyperphagia and a low BMR inherent to the syndrome. Hypothyroidism can cause arthralgia, lethargy, exertion fatigue, shortness of breath, and muscle problems [12,57]; this makes it hard to exercise, and increases the risk of obesity. Hypothyroidism is also directly associated with a decreased BMR, leading to a higher prevalence of obesity, which can further impair physical activity [58,59,60,61]. Obesity results in a high cardiovascular risk. Both indirect and direct cardiovascular effects of hypothyroidism make its early detection and treatment an important topic in this already vulnerable patient population [16].

#### 4.1.2. Brain Function

Thyroid function is responsible for a variety of physiological processes in the adult brain [62]. Adult-onset hypothyroidism can affect both cognitive function and psychological health [63]. Hypothyroidism can impair cognition, concentration, information processing speed, memory, perceptual function, and executive function [64,65]. Treatment with levothyroxine can reverse these symptoms [66]. Furthermore, anxiety and depressive symptoms are frequently reported in patients with hypothyroidism. These symptoms also improve after treatment with levothyroxine, leading to an increased quality of life [67,68].

The increased vulnerability of the patients, combined with diagnostic challenges and altered thyroid hormone metabolism, make the diagnosis and treatment of hypothyroidism an important issue in adults with PWS.

### 4.2. Diagnostic Challenges

Diagnostic challenges include patients’ delay, doctors’ delay, and unreliability of TSH.

#### 4.2.1. Patients’ Delay

Due to the intellectual disability that is often present in PWS, patients are often unable to express their complaints. Especially when the symptoms associated with hypothyroidism are subtle (e.g., mild fatigue or muscle weakness, or a slightly changed bowel pattern), they will not be reported by the patients.

#### 4.2.2. Doctors’ Delay

In the general population, TH concentrations are usually measured when there is a clinical suspicion of hypothyroidism. Unexplained weight gain, reduced appetite, fatigue, and constipation are well-known clinical signs of hypothyroidism that will alert most physicians to measure TH concentrations [12]. However, in patients with PWS, these symptoms are often unreliable. Unexplained weight gain will often be attributed to hyperphagia. In addition, this constant craving for food will make it easy to miss a slight reduction in appetite. Fatigue due to hypothyroidism can be easily mistaken for daytime sleepiness due to reduced hypothalamic arousal, which is often present in PWS [2,18]. Lastly, constipation is already present in 40% of patients with PWS, and will not alert physicians to screen for hypothyroidism [69].

#### 4.2.3. Unreliability of TSH

Apart from patients’ and doctors’ delay, there is another diagnostic challenge. Hypothyroidism can be both primary and central in PWS (Table 6). In our cohort, we also found that both central hypothyroidism (*n* = 12) and primary hypothyroidism (*n* = 3) were present. Serum TSH concentrations in patients with central hypothyroidism are often normal [70,71,72]. Furthermore, TSH can be affected by obesity [19,20,21,22]. Taken together, this means that TSH is less reliable in PWS. In our clinic, we have seen several examples of patients with untreated overt central hypothyroidism, which had been missed because the physician had only measured TSH and not fT4.

As symptoms of hypothyroidism are unreliable in patients with PWS, and hypothyroidism can be both primary and central, we recommend to screen for hypothyroidism by measuring serum TSH and fT4 concentrations every year.

### 4.3. Altered Thyroid Hormone Metabolism

Prescription of endocrine and non-endocrine medication may disturb TH metabolism. Likewise, altered levels of “hunger hormones” may affect TH concentrations. Examples of these TH metabolism-altering factors in adults with PWS include use of psychotropic drugs, growth hormone treatment, and disturbed leptin and ghrelin levels.

#### 4.3.1. Psychotropic Drugs

Psychotropic drugs can cause a disturbed synthesis and metabolism of TH [73,74]. Compared to the general endocrine population, the population of adults with PWS is characterized by frequent use of psychotropic drugs [75], such as antipsychotics, anxiolytics, and antidepressants. Psychotropic drugs can influence the synthesis and metabolism of TH in a variety of ways, such as changing iodine capture or decreasing thyrotropin-releasing hormone (TRH) responsiveness. Psychotropic drugs can also cause an altered deiodination of T4 to T3 by stimulating deiodinase activity [73,74,76]. The iodothyronine deiodinases D1, D2, and D3 regulate the conversion from the prohormone T4 (which is produced by the thyroid gland, and biologically inactive) to the active hormone T3. This conversion takes place mainly in peripheral tissues [77,78,79]. In our population, 40% of the patients used psychotropic drugs. T3 was significantly lower in patients who used psychotropic drugs than in patients who did not. However, patients who used psychotropic drugs were older (mean age 36 years) compared to patients who did not use psychotropic drugs (mean age 29 years), which might have influenced the results [23,24]. We did not find an association between specific types of psychotropic drugs and TH measurements. This could be related to a lack of power, as these subgroups were small.

#### 4.3.2. GH Treatment

GH treatment is often prescribed to children and young adults with PWS. In our population, one-third of the patients were treated with GH. Patients who used GH treatment had generally been receiving GH treatment for several years before visiting our outpatient clinic. It has been suggested that GH treatment enhances peripheral conversion of T4 to T3, resulting in decreased fT4 and increased T3 levels [80,81,82]. Several groups have studied the effect of GH treatment on TH levels, with contradictory results. Several studies showed that GH treatment does not induce hypothyroidism, but can unmask previously undiagnosed hypothyroidism in non-PWS individuals [83,84,85,86,87]. In children with PWS, fT4 concentrations decreased after the start of GH treatment, but remained within the low–normal range [25]. Two randomized controlled trials in adults with PWS showed no significant effect of GH treatment on fT4 and TSH [45,88], whereas one study showed increased T3 concentrations during GH treatment [45]. In our cohort, GH treatment was associated with higher T3 levels, while fT4 and TSH concentrations were similar for patients with and without GH treatment. To prevent the potential negative effects of missing ”unmasked” hypothyroidism, we recommend measuring TH concentrations 3–4 months after the start of GH treatment.

#### 4.3.3. Leptin and Ghrelin

High leptin levels caused by obesity in patients with PWS can lead to an increased conversion of T4 to T3 [89]. This mechanism might be partially responsible for the relatively low fT4 levels found in PWS patients [25,90]. Altered ghrelin levels in PWS [91,92] may impair the activity of the hypothalamic–pituitary–thyroid axis [93], which might increase the prevalence of hypothyroidism.

### 4.4. Strengths and Limitations

As with any study, our study has several strengths and limitations. One strength of our study is the population size, considering the rareness of the disease. Furthermore, we are the first to not only describe the prevalence of hypothyroidism and TH concentrations, but also the relationship with medication use. One of our limitations is that this was a retrospective study and, therefore, we had many missing values for T3, as this was not routinely measured in all patients. Another limitation is that thyroid peroxidase (TPO) antibodies were not measured; therefore, we were not able to distinguish autoimmune thyroid diseases. Furthermore, we did not measure thyroxine-binding globulin (TGB) and, therefore, we do not know whether free T3 levels were disturbed in our cohort. The last limitation is that laboratory measurements before the start of levothyroxine treatment were not available in 12 patients with hypothyroidism. In these cases we had to rely on referral letters that mentioned whether the patient had central or primary hypothyroidism.

## 5. Conclusions

In conclusion, one in six patients in our cohort of 122 adults with PWS had hypothyroidism, which is more frequent than in non-PWS adults. Hypothyroidism is often central in origin, and it is therefore important to measure not only TSH, but also fT4. We recommend yearly screening of fT4 and TSH to prevent the negative effects of untreated hypothyroidism on BMR, BMI, cardiovascular risk, and brain function. Additionally, we recommend measuring thyroid hormone concentrations 3–4 months after the start of GH treatment.

## Figures and Tables

**Table 1:** Baseline characteristics of 122 adults with Prader–Willi syndrome.

	Total
	*n* = 122
Age in years, median (IQR)	29 (21–39)
BMI in kg/m^2^, median (IQR)	29 (26–36)
Male gender, *n* (%)	58 (48%)
Genetic subtype	
Deletion, *n* (%)	66 (54%)
mUPD, *n* (%) ^a^	43 (35%)
ICD, *n* (%)	3 (2%)
Unknown, *n* (%)	10 (8%)
Growth hormone treatment	
Only during childhood, *n* (%)	12 (10%)
Only during adulthood, *n* (%)	3 (2%)
Both, *n* (%)	44 (36%)
Never, *n* (%)	63 (52%)
Current growth hormone treatment, *n* (%)	43 (35%)
Use of hydrocortisone	
Daily, *n* (%)	4 (3%)
During physical or psychological stress, *n* (%)	49 (40%)
Use of estrogen replacement therapy or oral contraceptives before screening, *n* (%)	34/64 females (53%)
Use of testosterone replacement therapy before screening, *n* (%)	24/58 males (41%)
Use of thyroid hormone replacement therapy before screening, *n* (%)	19 (16%)
Living situation	
With family, *n* (%)	31 (25%)
In a specialized PWS group home, *n* (%)	24 (20%)
In a non-specialized facility, *n* (%)	67 (55%)
Education level	
Secondary vocational education, *n* (%)	6 (5%)
Pre-vocational secondary education, *n* (%)	3 (2%)
Special education, *n* (%)	87 (71%)
No education, *n* (%)	5 (4%)
Unknown, *n* (%)	21 (17%)

Abbreviations: body mass index (BMI), paternal deletion (deletion), imprinting center defect (ICD), interquartile range (IQR), maternal uniparental disomy (mUPD), Prader–Willi syndrome (PWS). ^a^ In 14 patients with suspected mUPD, the parents were not available for genetic testing. Therefore, mUPD is the most likely genotype, but an ICD could not be ruled out in these patients.

**Table 2:** Prevalence of hypothyroidism and thyroid hormone levels in 122 patients with PWS (Part 1).

	Missing	Total *n* = 122	Males *n* = 58	Females *n* = 64	*p*-Value	Deletion *n* = 66	mUPD *n* = 43	*p*-Value
*n* of males, *n* of females	0	58, 64	58, 0	0, 64	NA	39, 37	21, 22	NA
Hypothyroidism, *n* (%)	0	21 (17%)	6 (10%)	15 (23%)	0.051	12 (18%)	7 (16%)	0.8
Subclinical hypothyroidism, *n* (%)	0	3 (2%)	0 (0%)	3 (5%)	NA	1 (2%)	2 (5%)	NA
Hyperthyroidism, *n* (%)	0	1 (1%)	0 (0%)	1 (2%)	NA	0 (0%)	0 (0%)	NA
*n* of males, *n* of females with normal thyroid function (*n* = 97)	0	52, 45	52, 0	0, 45	NA	26, 27	19, 15	NA
fT4 (pmol/L), median (IQR) (*n* = 97)	2	16.5 (14.3–18.5)	16.5 (14.6–18.5)	16.5 (14.1–18.6)	0.7	16.2 (14.1–17.7)	17.1 (14.8–18.9)	0.2
T3 (nmol/L), median (IQR) (*n* = 97)	52	1.9 (1.7–2.3)	1.8 (1.7–2.2)	2.1 (1.7–2.3)	0.5	2.0 (1.7–2.3)	1.7 (1.5–2.3)	0.2
TSH (mU/L), median (IQR) (*n* = 97)	0	1.6 (1.1–2.3)	1.5 (1.0–2.1)	1.9 (1.3–2.4)	0.06	1.7 (1.4–2.3)	1.5 (1.0–2.3)	0.06

Abbreviations: paternal deletion (deletion), free thyroxine (fT4), interquartile range (IQR), maternal uniparental disomy (mUPD), not applicable (NA), triiodothyronine (T3), thyroid-stimulating hormone (TSH). Laboratory concentrations are for patients with normal thyroid function only (*n* = 97).

**Table 3:** Prevalence of hypothyroidism and thyroid hormone levels in 122 patients with PWS (Part 2).

	Age < 25 years *n* = 47	Age 25–30 years *n* = 22	Age > 30 years *n* = 53	*p*-Value	BMI < 25 kg/m^2^ *n* = 25	BMI 25–30 kg/m^2^ *n* = 45	BMI > 30 kg/m^2^ *n* = 52	*p*-Value
*n* of males, *n* of females	21, 26	9, 13	28, 25	NA	12, 13	29, 16	17, 35	NA
Hypothyroidism, *n* (%)	10 (21%)	6 (27%)	5 (9%)	0.4	5 (20%)	8 (18%)	8 (15%)	0.6
Subclinical hypothyroidism, *n* (%)	1 (2%)	1 (5%)	1 (2%)	NA	0 (0%)	1 (2%)	2 (4%)	NA
Hyperthyroidism, *n* (%)	0 (0%)	0 (0%)	1 (2%)	NA	0 (0%)	1 (2%)	0 (0%)	NA
*n* of males, *n* of females with normal thyroid function (*n* = 97)	18, 18	8, 7	26, 20	NA	11, 9	25, 10	16, 26	NA
fT4 (pmol/L), median (IQR) (*n* = 97)	16.5 (14.9–18.1)	15.4 (14.0–19.1)	16.7 (14.1–18.9)	0.2	15.9 (14.6–18.2)	16.6 (14.2–18.8)	16.7 (14.2–18.4)	1
T3 (nmol/L), median (IQR) (*n* = 97)	2.1 (1.8–2.2)	2.2 (1.8–2.5)	1.7 (1.6–1.9)	0.003	2.3 (1.9–2.4)	1.7 (1.6–2.3)	1.9 (1.7–2.2)	1
TSH (mU/L), median (IQR) (*n* = 97)	1.6 (1.2–2.2)	1.7 (1.4–2.7)	1.5 (0.9–2.3)	0.6	1.6 (1.2–2.2)	1.4 (0.9–2.3)	1.8 (1.3–2.7)	0.3

Abbreviations: body mass index (BMI), free thyroxine (fT4), interquartile range (IQR), not applicable (NA), triiodothyronine (T3), thyroid-stimulating hormone (TSH). *P*-values are calculated with age and BMI as continuous variables. Laboratory measurements are for patients with normal thyroid function only (*n* = 97).

**Table 4:** Prevalence of hypothyroidism and thyroid hormone levels in 122 patients with PWS (Part 3).

	Current GH Treatment *n* = 43	No Current GH Treatment *n* = 79	*p*-Value	Psychotropic Drugs *n* = 49	No Psychotropic Drugs *n* = 73	*p*-Value
*n* of males, *n* of females	19, 24	39, 40	NA	25, 24	33, 40	NA
Hypothyroidism, *n* (%)	8 (19%)	13 (16%)	0.8	10 (20%)	11 (15%)	0.5
Subclinical hypothyroidism, *n* (%)	2 (5%)	1 (1%)	NA	1 (2%)	2 (3%)	NA
Hyperthyroidism, *n* (%)	0 (0%)	1 (1%)	NA	0 (0%)	1 (1%)	NA
*n* of males, *n* of females with normal thyroid function (*n* = 97)	17, 16	35, 29	NA	21, 17	31, 28	NA
fT4 (pmol/L), median (IQR) (*n* = 97)	16.0 (14.3–18.0)	16.9 (14.5–18.8)	0.3	16.6 (14.7–18.7)	16.3 (14.1–18.1)	0.8
T3 (nmol/L), median (IQR) (*n* = 97)	2.1 (1.8–2.4)	1.8 (1.6–2.2)	0.03	1.7 (1.6–2.0)	2.1 (1.7–2.3)	0.02
TSH (mU/L), median (IQR) (*n* = 97)	1.6 (1.2–2.0)	1.6 (1.0–2.3)	0.8	1.5 (0.9–2.5)	1.7 (1.2–2.2)	0.7

Abbreviations: free thyroxine (fT4), growth hormone (GH), interquartile range (IQR), not applicable (NA), triiodothyronine (T3), thyroid-stimulating hormone (TSH). Laboratory measurements are for patients with normal thyroid function only (*n* = 97).

**Table 5:** Patient characteristics of cohorts assessed by previous studies (Part 1).

	Article	*n*	Country	Age Range (years)	Genotype (Deletion, mUPD, ICD, Translocation)	Gender	Mean BMI (kg/m^2^)	Current GH Treatment
**Children**	Tauber et al. (2000) [33]	28	France	-	36%, 14%, 0%, 0% (25% NA, 18% no genetic analysis, 7% no genetic abnormality)	12 M, 16 F	-	50%
	Festen et al. (2007) [25]	75	The Netherlands	Median (IQR): 4.7 (2.7–7.6)	35%, 32%, 9%, 1% (23% NA)	39 M, 36 F	Median (IQR): 18 (16–20)	0%
	Vaiani et al. (2010) [34]	18	Argentina	0–2	61%, 28%, 0%, 0% (11% NA)	11 M, 7 F	-	-
	Wong et al. (2010) [35]	20	USA	Mean ± SD: 4.0 ± 0.8	-	12 M, 8 F	20	0%
	Diene et al. (2010) [36]	127	France	0–18 ^a^	63%, 25%, 2%, 1% (1% other, 8% NA) ^a^	77 M, 65 F ^a^	Median BMI Z-score +1.3 ^a^	87% ^a^
	Sharkia et al. (2013) [37]	31 ^b^ TRH-ST: 21 Neonates: 23	Canada	TRH-ST: 0–18 Neonates: 0	TRH-ST: 62%, 29%, 0%, 0% (10% NA) Neonates: 43%, 48%, 0%, 0% (9% NA)	TRH-ST: 7 M, 14 F Neonates: 9 M, 14 F	TRH-ST: mean BMI Z-score +1.2 Neonates: NA	TRH-ST: 86% Neonates: 0%
	Kim et al. (2014) [38]	14	Korea	0–3 ^c^	93%, 7%, 0%, 0% ^c^	16 M, 14 F ^c^	BMI-SDS: 0.66	0%
	Iughetti et al. (2019) [31]	243	Italy	0–18	57%, 34%, 0%, 1% (8% NA) ^d^	233 M, 106 F ^d^	20	27%
	Oto et al. (2020) [39]	51	Japan	0–7	61%, 39%, 0%, 0%	29 M, 22 F	-	-
	Lu et al. (2020) [40]	48	China	0–15	77%, 23%, 0%, 0%	32 M, 16 F	Mean BMI Z-score: 0.8	0%
	Konishi et al. (2020) [41]	43	Japan	0–3	60%, 40%, 0%, 0%	17 M, 26 F	Median BMI-SDS: -1.47	0%
	Dağdeviren Çakır et al. (2021) [42]	52	Turkey	0–15	69%, 12%, 2%, 0% (17% NA)	26 M, 26 F	20	40%
**Children and Adults**	Höybye et al. (2002) [43]	13 ^e^	Sweden	17–37	-	7 M, 6 F	35	-
	Butler et al. (2007) [32]	47	USA	10–44	55%, 45%, 0%, 0%	21 M, 26 F	34	0%
	Miller et al. (2008) [44]	27	USA	0–39	74%, 26%, 0%, 0%	17 M, 10 F	Obesity: 74%	0%
	Mogul et al. (2008) [45]	38	USA	17–49	-	13 M, 25 F	35	0% ^f^
	Farholt et al. (2011) [46]	65	Denmark	0–48	65%, 20%, 3%, 0% (12% NA)	33 M, 32 F	Median BMI-SDS: 0.92	62%
	Laurier et al. (2015) [47]	154	France	16–54	66%, 16%, 2%, 2% (15% NA)	68 M, 86 F	42	14%
	Coupaye et al. (2016) [48] ^g^	73	France	16–58	64%, 36%, 0%, 0%	35 M, 38 F	Deletion: 41, mUPD: 35	15%
	Proffitt et al. (2019) [49]	2029	USA	0–84	42%, 19%, 2%, 0% (37% NA)	934 M, 1000 F	Living: 29, deceased: 52	Living: 51%, deceased: 22%
	Pemmasani et al. (2021) [50]	480	USA	Mean ± SD: 27 ± 19	-	242 M, 238 F	Obesity: 41%	-
**Adults**	Van Nieuwpoort et al. (2011) [51,52] ^h^	15	The Netherlands	19–42	93%, 7%, 0%, 0%	4 M, 11 F	Median: 28	0%
	Sinnema et al. (2011) [52] ^h^	102	The Netherlands	18–66	54%, 43%, 3%, 0%	49 M, 53 F	32	5%
	Grugni et al. (2013) [53] ^i^	108	Italy	18–43	68%, 25%, 0%, 2% (6% NA)	47 M, 61 F	Median in non-obese: 26, median in obese: 45	-
	Iughetti et al. (2019) [31] ^i^	96	Italy	19–50	57%, 34%, 0%, 1% (8% NA) ^d^	233 M, 106 F ^d^	43	-
	Radetti et al. (2020) [54] ^i^	120	Italy	18–59	71%, 28%, 0%, 0% (2% NA)	69 M, 51 F	37	20%

Abbreviations: body mass index (BMI), deletion (paternal deletion), females (F), growth hormone (GH), ICD (imprinting center defect), interquartile range (IQR), males (M), mUPD (maternal uniparental disomy), not available (NA or -), standard deviation (SD), standard deviation score (SDS), thyrotropin-releasing hormone stimulation test group (TRH-ST), United States of America (USA). ^a^ Data for the whole cohort of 142 patients; however, hypothyroidism was only assessed in 127 patients. ^b^ Thirteen patients were included in both the TRH-ST group and the neonate group. ^c^ Data for the whole cohort of 30 patients; however, thyroid hormone values were only given for 14 patients. ^d^ Data for the whole cohort of children and adults. ^e^ Only data for methylation-positive subjects are included in the table. ^f^ All patients were growth hormone deficient and growth hormone treatment had not been started yet. ^g^ A more recent study conducted by the same research group (Paepegaey et al. 2018 [55]) with a larger study population was available. As this study population was largely the same as that of Coupaye et al. and this study did not report any laboratory values, it was not included in the table. Paepegaey et al. evaluated thyroid function in 91 adults, of whom 29 had hypothyroidism (31%). ^h^ Van Nieuwpoort et al. (2011) performed a systematic screening with blood measurements in patients who might also have been described in Sinnema et al. (2011), where data were collected based on questionnaires and medical histories. ^i^ The study population of Grugni et al. (2013) contains 30 patients who were not described in Iughetti et al. (2019) and/or Radetti et al. (2020). The study population of Radetti et al. (2020) contains 36 subjects with PWS who were not described in Iughetti et al. (2019).

**Table 6:** Patient characteristics of cohorts assessed by previous studies (Part 2).

	Article	Total Overt Hypothyroidism (Central, Primary Hypothyroidism) (%)	Subclinical Hypothyroidism (%)	TSH (mU/L)	Free T4 (pmol/L)
**Children**	Tauber et al. (2000) [33]	32	-	-	Mean ± SD: 8.1 ± 1.1 pg/mL *Mean: 10 nmol/L*
	Festen et al. (2007) [25]	6 ^a^	-	Median (IQR): 2.0 (1.6–2.7) mU/L	Median (IQR): 16.2 (14.3–17.8) pmol/L
	Vaiani et al. (2010) [34]	72 ^b^	-	Median (range): TAD: 1.4 (0.8–5.7) mU/L NTAD: 2.9 (1.4–5.3) mU/L	Median (range): TAD: 9.1 (2.6–11.8) pmol/L NTAD: 12.9 (12.1–21.0) pmol/L
	Wong et al. (2010) [35]	0	-	-	-
	Diene et al. (2010) [36]	24	-	-	-
	Sharkia et al. (2013) [37]	TRH-ST: 5 ^c^ Neonates: 0 ^e^	-	Mean ± SD (range) TRH-ST: 1.9 ± 1.0 (0.8–4.2) mU/L ^d^ Neonates: 3.1 ± 2.3 (0.4–10.0) mU/L ^e^	Mean ± SD (range) TRH-ST: 10.4 ± 1.1 (8.2–13.5) pmol/L ^d^
	Kim et al. (2014) [38]	-	-	Mean ± SD: 3.2 ± 2.1 mU/L	Mean ± SD: 1.1 ± 0.2 ng/dL *Mean: 14 pmol/L*
	Iughetti et al. (2019) [31]	11 (8, 2) ^f^	5	Mean ± SD (median): 2.7 ± 2.1 (2.2) mU/L	Mean ± SD (median): 10.6 ± 2.2 (10.3) pg/mL *Mean: 13.6 pmol/L, median: 13.3 pmol/L*
	Oto et al. (2020) [39]	TRH-ST: 4 ^c^	-	Median (IQR): 2.3 (1.2–3.6) mU/L	Median (IQR): 1.18 (1.02–1.24) ng/dL *Median (IQR): 15 (13 – 16) pmol/L*
	Lu et al. (2020) [40]	-	-	-	≤2 years old: mean ± SD: 0.7 ± 0.2 ng/dL >2 years old: mean ± SD: 0.9 ± 0.2 ng/dL *≤2 years old: mean: 9 pmol/L* *>2 years old: mean: 12 pmol/L*
	Konishi et al. (2020) [41]	30	-	Median (IQR): 2.4 (1.8–3.4) mU/L	Median (IQR): 11.6 (9.9–14.0) pmol/L
	Dağdeviren Çakır et al. (2021) [42]	33 (31, 2)	-	-	-
**Children and Adults**	Höybye et al. (2002) [43]	0	-	-	-
	Butler et al. (2007) [32]	2 (2, 0)	-	Mean ± SD: 2.2 ± 1.3 mU/L	Mean ± SD: 1.1 ± 0.2 ng/dL (*n* = 43) *Mean: 14 pmol/L*
	Miller et al. (2008) [44]	NA (19, NA) ^g^	-	-	-
	Mogul et al. (2008) [45]	0	-	Mean ± SD (range): 1.5 ± 0.2 (0.01–7.7) mU/L	Mean ± SD (range): 1.1 ± 0.04 (0.6–1.7) ng/dL *Mean (range): 14 (8–22) pmol/L*
	Farholt et al. (2011) [46]	5	-	-	-
	Laurier et al. (2015) [47]	26	-	-	-
	Coupaye et al. (2016) [48]	26 ^h^	-	-	Mean ± SD: Deletion: 14.0 ± 2.0 pmol/L UPD: 15.1 ± 2.7 pmol/L
	Proffitt et al. (2019) [49]	9	-	-	-
	Pemmasani et al. (2021) [50]	16	-	-	-
**Adults**	Van Nieuwpoort et al. (2011) [51]	13 ^i^	-	Median (IQR): 2.3 (1.85) mU/L	Median (IQR): 15.4 (1.9) pmol/L
	Sinnema et al. (2011) [52]	9	-	-	-
	Grugni et al. (2013) [53]	5	-	-	-
	Iughetti et al. (2019) [31]	6 (4, 2)	1	Mean ± SD (median): 2.2 ± 1.4 (2.0) mU/L	Mean ± SD (median): 11.4 ± 2.0 (11.2) pg/mL *Mean: 15 pmol/L, median: 14 pmol/L*
	Radetti et al. (2020) [54]	10	-	-	-

Abbreviations: interquartile range (IQR), not available (NA or -), non-thyroid axis dysfunction (NTAD), standard deviation (SD), thyroxine (T4), thyroid axis dysfunction (TAD), thyrotropin-releasing hormone stimulation test (TRH-ST), thyroid-stimulating hormone (TSH). When laboratory measurements were reported in non-SI units, the converted values are shown in *italics*. Total overt hypothyroidism is the sum of central, primary, and congenital hypothyroidism. ^a^ Five of 79 patients had free T4 levels below -2 standard deviation score; however, thyroid hormone levels of 4 of these patients are reported separately, as they already received thyroid hormone replacement therapy. ^b^ Percentage of thyroid axis dysfunction based on a free T4 or total T4 level below the 2.5th percentile. ^c^ Based on TSH response to thyrotropin-releasing hormone. ^d^ For patients with a normal thyrotropin-releasing hormone test only. ^e^ Based on blood samples collected on filter paper for the universal newborn screening for congenital hypothyroidism. ^f^ Congenital hypothyroidism in 2%. ^g^ Only central hypothyroidism was evaluated, and was present in 19%. Additionally, one patient had previously been diagnosed with autoimmune primary hypothyroidism. ^h^ A more recent study from the same research group (Paepegaey et al. 2018 [55]) with a larger study population was available. As this study population was largely the same as that of Coupaye et al., and this study did not report any laboratory values, it was not included in the table. Paepegaey et al. evaluated thyroid function in 91 adults, of whom 29 had hypothyroidism (31%). ^i^ Central in origin according to the patients. Additionally, one patient had hyperthyroidism.

**Table 7:** Patient characteristics of cohorts assessed by previous studies (Part 3).

	Article	Total T4 (nmol/L)	Free T3 (pmol/L)	Total T3 (nmol/L)	Reverse T3 (nmol/L)
**Children**	Tauber et al. (2000) [33]	-	-	Mean ± SD: 118 ± 3.1 ng/dL *Mean: 1.8 pmol/L*	-
	Festen et al. (2007) [25]	Median (IQR): 98.0 (85.3–113) nmol/L	-	Median (IQR): 2.6 (2.3–3.0) nmol/L	Median (IQR): 0.3 (0.3–0.4) nmol/L
	Vaiani et al. (2010) [34]	Median (range): TAD: 88.8 (57.9–109) nmol/L NTAD: 112 (86.2–126) nmol/L	-	Median (range): TAD: 2.5 (1.4–3.2) nmol/L NTAD: 2.4 (2.3–3.0) nmol/L	-
	Wong et al. (2010) [35]	-	-	-	-
	Diene et al. (2010) [36]	-	-	-	-
	Sharkia et al. (2013) [37]	- -	Mean ± SD (range) TRH-ST: 6.1 ± 1.0 (4.8–8.4) pmol/L ^a^	-	-
	Kim et al. (2014) [38]	-	-	-	-
	Iughetti et al. (2019) [31]	-	Mean ± SD (median): 3.7 ± 1.0 (3.6) pg/mL *Mean: 5.7 pmol/L, median: 5.5 pmol/L*	-	-
	Oto et al. (2020) [39]	-	Median (IQR): 4.0 (3.5–4.4) pg/mL *Median (IQR): 6.2 (5.4–6.8) pmol/L*	-	-
	Lu et al. (2020) [40]	≤2 years old: mean ± SD: 7.5 ± 1.7 µg/dL >2 years old: mean ± SD: 9.0 ± 2.5 µg/dL *≤2 years old: mean: 96.5 nmol/L* *>2 years old: mean: 116 nmol/L*	-	-	-
	Konishi et al. (2020) [41]	-	Median (IQR): 4.8 (4.1–5.6) pmol/L	-	-
	Dağdeviren Çakır et al. (2021) [42]	-	-	-	-
**Children and Adults**	Höybye et al. (2002) [43]	-	-	-	-
	Butler et al. (2007) [32]	Mean ± SD: 8.1 ± 2.0 μg/dL (*n* = 38) *Mean: 104 nmol/L*	-	Mean ± SD: 137 ± 38 ng/dL(*n* = 41) *Mean: 2.1 nmol/L*	-
	Miller et al. (2008) [44]	-	-	-	-
	Mogul et al. (2008) [45]	Mean ± SD (range): 8.6 ± 0.3 (4.2–14.4) μg/dL *Mean (range): 111 (54–185) nmol/L*	-	Mean ± SD (range): 131 ± 8 (46–251) ng/dL *Mean (range): 2.0 (0.7–3.9) nmol/L*	-
	Farholt et al. (2011) [46]	-	-	-	-
	Laurier et al. (2015) [47]	-	-	-	-
	Coupaye et al. (2016) [48]	-	-	-	-
	Proffitt et al. (2019) [49]	-	-	-	-
	Pemmasani et al. (2021) [50]	-	-	-	-
**Adults**	Van Nieuwpoort et al. (2011) [51]	-	Median (IQR): 5.1 (0.8) pmol/L	-	-
	Sinnema et al. (2011) [52]	-	-	-	-
	Grugni et al. (2013) [53]	-	-	-	-
	Iughetti et al. (2019) [31]	-	Mean ± SD (median): 3.1 ± 0.7 (3.0) pg/mL *Mean: 4.7 pmol/L, median: 4.6 pmol/L*	-	-
	Radetti et al. (2020) [54]	-	-	-	-

Abbreviations: interquartile range (IQR), not available (-), non-thyroid axis dysfunction (NTAD), standard deviation (SD), triiodothyronine (T3), thyrotropin-releasing hormone stimulation test (TRH-ST), thyroid axis dysfunction (TAD). When laboratory measurements were reported in non-SI units, the converted values are shown in *italics*. ^a^ For patients with a normal TRH-ST only.

## Data Availability

The datasets generated during and/or analyzed during the current study are not publicly available, in order to protect the privacy of the patients participating in this study. As Prader–Willi syndrome is a rare syndrome, individual patient data could be traced back to the individual.

## Supplementary Materials

The following are available online at https://www.mdpi.com/article/10.3390/jcm10173804/s1: Table S1: Full search strategy (Embase); Table S2: Prevalence of hypothyroidism and thyroid hormone levels in relation to use of psychotropic drugs (Part 1); Table S3. Prevalence of hypothyroidism and thyroid hormone levels in relation to use of psychotropic drugs (Part 2); Figure S1: Scatterplot of T3, fT4, and TSH in relation to BMI for patients who use psychotropic drugs versus patients who do not; Figure S2: Scatterplot of T3, fT4, and TSH in relation to BMI for patients who currently use growth hormone treatment versus patients who do not.

## References

[1] Cassidy S.B., Schwartz S., Miller J.L., Driscoll D.J. (2012). Prader-Willi syndrome. Genet. Med..

[2] Cassidy S.B., Driscoll D.J. (2009). Prader-Willi syndrome. Eur. J. Hum. Genet..

[3] Cheon C.K. (2016). Genetics of Prader-Willi syndrome and Prader-Will-Like syndrome. Ann. Pediatric Endocrinol. Metab..

[4] Mackay J., McCallum Z., Ambler G.R., Vora K., Nixon G., Bergman P., Shields N., Milner K., Kapur N., Crock P. (2019). Requirements for improving health and well-being of children with Prader-Willi syndrome and their families. J. Paediatr. Child. Health.

[5] Muscogiuri G., Formoso G., Pugliese G., Ruggeri R.M., Scarano E., Colao A. (2019). Prader- Willi syndrome: An uptodate on endocrine and metabolic complications. Rev. Endocr. Metab. Disord..

[6] Angulo M.A., Butler M.G., Cataletto M.E. (2015). Prader-Willi syndrome: A review of clinical, genetic, and endocrine findings. J. Endocrinol. Invest..

[7] Swaab D.F. (1997). Prader-Willi syndrome and the hypothalamus. Acta Paediatr..

[8] Burman P., Ritzen E.M., Lindgren A.C. (2001). Endocrine dysfunction in Prader-Willi syndrome: A review with special reference to GH. Endocr. Rev..

[9] Holm V.A., Cassidy S.B., Butler M.G., Hanchett J.M., Greenswag L.R., Whitman B.Y., Greenberg F. (1993). Prader-Willi syndrome: Consensus diagnostic criteria. Pediatrics.

[10] De Geronimo V., Cannarella R., La Vignera S. (2021). Thyroid Function and Obesity: From Mechanisms to the Benefits of Levothyroxine in Obese Patient. Endocr. Metab. Immune Disord. Drug Targets.

[11] Akici N., Onal Z.E., Gurbuz T., Sag C., Kilinc S. (2020). Atherogenic Indices in the Assessment of Cardiovascular Disease Risk in Children with Obesity and Subclinical Hypothyroidism. Acta Endocrinol..

[12] Chaker L., Bianco A.C., Jonklaas J., Peeters R.P. (2017). Hypothyroidism. Lancet.

[13] Butler M.G., Manzardo A.M., Heinemann J., Loker C., Loker J. (2017). Causes of death in Prader-Willi syndrome: Prader-Willi Syndrome Association (USA) 40-year mortality survey. Genet. Med..

[14] Pacoricona Alfaro D.L., Lemoine P., Ehlinger V., Molinas C., Diene G., Valette M., Pinto G., Coupaye M., Poitou-Bernert C., Thuilleaux D. (2019). Causes of death in Prader-Willi syndrome: Lessons from 11 years’ experience of a national reference center. Orphanet J. Rare Dis..

[15] Whittington J.E., Holland A.J., Webb T., Butler J., Clarke D., Boer H. (2001). Population prevalence and estimated birth incidence and mortality rate for people with Prader-Willi syndrome in one UK Health Region. J. Med. Genet..

[16] Klein I., Danzi S. (2016). Thyroid Disease and the Heart. Curr. Probl. Cardiol..

[17] Rotondi M., Magri F., Chiovato L. (2010). Risk of coronary heart disease and mortality for adults with subclinical hypothyroidism. JAMA.

[18] Pellikaan K., Rosenberg A.G.W., Kattentidt-Mouravieva A.A., Kersseboom R., Bos-Roubos A.G., Veen-Roelofs J.M.C., van Wieringen N., Hoekstra F.M.E., van den Berg S.A.A., van der Lely A.J. (2020). Missed Diagnoses and Health Problems in Adults With Prader-Willi Syndrome: Recommendations for Screening and Treatment. J. Clin. Endocrinol. Metab..

[19] Sanyal D., Raychaudhuri M. (2016). Hypothyroidism and obesity: An intriguing link. Indian J. Endocrinol. Metab..

[20] Wang Y., Dong X., Fu C., Su M., Jiang F., Xu D., Li R., Qian J., Wang N., Chen Y. (2020). Thyroid Stimulating Hormone (TSH) Is Associated With General and Abdominal Obesity: A Cohort Study in School-Aged Girls During Puberty in East China. Front. Endocrinol..

[21] Chen X., Deng S., Sena C., Zhou C., Thaker V.V. (2021). Relationship of TSH Levels with Cardiometabolic Risk Factors in US Youth and Reference Percentiles for Thyroid Function. J. Clin. Endocrinol. Metab..

[22] Knudsen N., Laurberg P., Rasmussen L.B., Bulow I., Perrild H., Ovesen L., Jorgensen T. (2005). Small differences in thyroid function may be important for body mass index and the occurrence of obesity in the population. J. Clin. Endocrinol. Metab..

[23] Waring A.C., Arnold A.M., Newman A.B., Buzkova P., Hirsch C., Cappola A.R. (2012). Longitudinal changes in thyroid function in the oldest old and survival: The cardiovascular health study all-stars study. J. Clin. Endocrinol. Metab..

[24] Bremner A.P., Feddema P., Leedman P.J., Brown S.J., Beilby J.P., Lim E.M., Wilson S.G., O’Leary P.C., Walsh J.P. (2012). Age-related changes in thyroid function: A longitudinal study of a community-based cohort. J. Clin. Endocrinol. Metab..

[25] Festen D.A., Visser T.J., Otten B.J., Wit J.M., Duivenvoorden H.J., Hokken-Koelega A.C. (2007). Thyroid hormone levels in children with Prader-Willi syndrome before and during growth hormone treatment. Clin. Endocrinol..

[26] Michalaki M.A., Vagenakis A.G., Leonardou A.S., Argentou M.N., Habeos I.G., Makri M.G., Psyrogiannis A.I., Kalfarentzos F.E., Kyriazopoulou V.E. (2006). Thyroid function in humans with morbid obesity. Thyroid.

[27] Ambrosi B., Masserini B., Iorio L., Delnevo A., Malavazos A.E., Morricone L., Sburlati L.F., Orsi E. (2010). Relationship of thyroid function with body mass index and insulin-resistance in euthyroid obese subjects. J. Endocrinol. Invest..

[28] Kitahara C.M., Platz E.A., Ladenson P.W., Mondul A.M., Menke A., de Gonzalez A.B. (2012). Body fatness and markers of thyroid function among U.S. men and women. PLoS ONE.

[29] Fu J., Zhang L., An Y., Duan Y., Liu J., Wang G. (2021). Association Between Body Mass Index and Thyroid Function in Euthyroid Chinese Adults. Med. Sci Monit.

[30] Meng Z., Liu M., Zhang Q., Liu L., Song K., Tan J., Jia Q., Zhang G., Wang R., He Y. (2015). Gender and Age Impacts on the Association Between Thyroid Function and Metabolic Syndrome in Chinese. Medicine.

[31] Iughetti L., Vivi G., Balsamo A., Corrias A., Crino A., Delvecchio M., Gargantini L., Greggio N.A., Grugni G., Hladnik U. (2019). Thyroid function in patients with Prader-Willi syndrome: An Italian multicenter study of 339 patients. J. Pediatric Endocrinol. Metab..

[32] Butler M.G., Theodoro M., Skouse J.D. (2007). Thyroid function studies in Prader-Willi syndrome. Am. J. Med. Genet. A.

[33] Tauber M., Barbeau C., Jouret B., Pienkowski C., Malzac P., Moncla A., Rochiccioli P. (2000). Auxological and endocrine evolution of 28 children with Prader-Willi syndrome: Effect of GH therapy in 14 children. Horm. Res. Paediatr..

[34] Vaiani E., Herzovich V., Chaler E., Chertkoff L., Rivarola M.A., Torrado M., Belgorosky A. (2010). Thyroid axis dysfunction in patients with Prader-Willi syndrome during the first 2 years of life. Clin. Endocrinol..

[35] Wong K., Levitsky L.L., Misra M. (2010). Predictors and growth consequences of central hypothyroidism in pediatric patients receiving recombinant human growth hormone. J. Pediatric Endocrinol. Metab..

[36] Diene G., Mimoun E., Feigerlova E., Caula S., Molinas C., Grandjean H., Tauber M., French Reference Centre for PWS (2010). Endocrine disorders in children with Prader-Willi syndrome--data from 142 children of the French database. Horm. Res. Paediatr..

[37] Sharkia M., Michaud S., Berthier M.T., Giguere Y., Stewart L., Deladoey J., Deal C., Van Vliet G., Chanoine J.P. (2013). Thyroid function from birth to adolescence in Prader-Willi syndrome. J. Pediatrics.

[38] Kim Y.J., Cheon C.K. (2014). Prader-Willi syndrome: A single center’s experience in Korea. Korean J. Pediatrics.

[39] Oto Y., Murakami N., Matsubara K., Saima S., Ogata H., Ihara H., Nagai T., Matsubara T. (2020). Effects of growth hormone treatment on thyroid function in pediatric patients with Prader-Willi syndrome. Am. J. Med. Genet. A.

[40] Lu A., Luo F., Sun C., Zhang X., Wang L., Lu W. (2020). Sleep-disordered breathing and genetic findings in children with Prader-Willi syndrome in China. Ann. Transl. Med..

[41] Konishi A., Ida S., Shoji Y., Etani Y., Kawai M. (2020). Central hypothyroidism improves with age in very young children with Prader-Willi syndrome. Clin. Endocrinol..

[42] Dagdeviren Cakir A., Bas F., Akin O., Siklar Z., Ozcabi B., Berberoglu M., Kardelen A.D., Bayramoglu E., Poyrazoglu S., Aydin M. (2021). Clinical Characteristics and Growth Hormone Treatment in Patients with Prader-Willi Syndrome. J. Clin. Res. Pediatric Endocrinol..

[43] Hoybye C., Hilding A., Jacobsson H., Thoren M. (2002). Metabolic profile and body composition in adults with Prader-Willi syndrome and severe obesity. J. Clin. Endocrinol. Metab..

[44] Miller J.L., Goldstone A.P., Couch J.A., Shuster J., He G., Driscoll D.J., Liu Y., Schmalfuss I.M. (2008). Pituitary abnormalities in Prader-Willi syndrome and early onset morbid obesity. Am. J. Med. Genet. A.

[45] Mogul H.R., Lee P.D., Whitman B.Y., Zipf W.B., Frey M., Myers S., Cahan M., Pinyerd B., Southren A.L. (2008). Growth hormone treatment of adults with Prader-Willi syndrome and growth hormone deficiency improves lean body mass, fractional body fat, and serum triiodothyronine without glucose impairment: Results from the United States multicenter trial. J. Clin. Endocrinol. Metab..

[46] Farholt S., Sode-Carlsen R., Christiansen J.S., Østergaard J.R., Høybye C. (2011). Normal cortisol response to high-dose synacthen and insulin tolerance test in children and adults with Prader-Willi syndrome. J. Clin. Endocrinol. Metab..

[47] Laurier V., Lapeyrade A., Copet P., Demeer G., Silvie M., Bieth E., Coupaye M., Poitou C., Lorenzini F., Labrousse F. (2015). Medical, psychological and social features in a large cohort of adults with Prader-Willi syndrome: Experience from a dedicated centre in France. J. Intellect. Disabil. Res..

[48] Coupaye M., Tauber M., Cuisset L., Laurier V., Bieth E., Lacorte J.M., Oppert J.M., Clement K., Poitou C. (2016). Effect of Genotype and Previous GH Treatment on Adiposity in Adults With Prader-Willi Syndrome. J. Clin. Endocrinol. Metab..

[49] Proffitt J., Osann K., McManus B., Kimonis V.E., Heinemann J., Butler M.G., Stevenson D.A., Gold J.A. (2019). Contributing factors of mortality in Prader-Willi syndrome. Am. J. Med. Genet. A.

[50] Pemmasani G., Yandrapalli S. (2021). Age-stratified prevalence of relevant comorbidities and etiologies for hospitalizations in Prader–Willi syndrome patients. Am. J. Med. Genet. Part A.

[51] van Nieuwpoort I.C., Sinnema M., Castelijns J.A., Twisk J.W., Curfs L.M., Drent M.L. (2011). The GH/IGF-I axis and pituitary function and size in adults with Prader-Willi syndrome. Horm. Res. Paediatr..

[52] Sinnema M., Maaskant M.A., van Schrojenstein Lantman-de Valk H.M., van Nieuwpoort I.C., Drent M.L., Curfs L.M., Schrander-Stumpel C.T. (2011). Physical health problems in adults with Prader-Willi syndrome. Am. J. Med. Genet. A.

[53] Grugni G., Crino A., Bedogni G., Cappa M., Sartorio A., Corrias A., Di Candia S., Gargantini L., Iughetti L., Pagano C. (2013). Metabolic syndrome in adult patients with Prader-Willi syndrome. Nutr. Metab. Cardiovasc. Dis..

[54] Radetti G., Fanolla A., Lupi F., Sartorio A., Grugni G. (2020). Accuracy of different indexes of body composition and adiposity in identifying metabolic syndrome in adult subjects with Prader-Willi syndrome. J. Clin. Med..

[55] Paepegaey A.C., Coupaye M., Jaziri A., Ménesguen F., Dubern B., Polak M., Oppert J.M., Tauber M., Pinto G., Poitou C. (2018). Impact of transitional care on endocrine and anthropometric parameters in Prader-Willi syndrome. Endocr. Connect..

[56] Garmendia Madariaga A., Santos Palacios S., Guillen-Grima F., Galofre J.C. (2014). The incidence and prevalence of thyroid dysfunction in Europe: A meta-analysis. J. Clin. Endocrinol. Metab..

[57] Kaminsky P., Robin-Lherbier B., Brunotte F., Escanye J.M., Walker P., Klein M., Robert J., Duc M. (1992). Energetic metabolism in hypothyroid skeletal muscle, as studied by phosphorus magnetic resonance spectroscopy. J. Clin. Endocrinol. Metab..

[58] Yavuz S., Del Prado S.S.N., Celi F.S. (2019). Thyroid Hormone Action and Energy Expenditure. J. Endocr. Soc..

[59] Butler M.G., Theodoro M.F., Bittel D.C., Donnelly J.E. (2007). Energy expenditure and physical activity in Prader-Willi syndrome: Comparison with obese subjects. Am. J. Med. Genet. A.

[60] Schoeller D.A., Levitsky L.L., Bandini L.G., Dietz W.W., Walczak A. (1988). Energy expenditure and body composition in Prader-Willi syndrome. Metabolism.

[61] Biondi B. (2010). Thyroid and obesity: An intriguing relationship. J. Clin. Endocrinol. Metab..

[62] Bernal J. (2005). Thyroid hormones and brain development. Vitam. Horm..

[63] Samuels M.H. (2014). Psychiatric and cognitive manifestations of hypothyroidism. Curr. Opin. Endocrinol. Diabetes Obes..

[64] Correia N., Mullally S., Cooke G., Tun T.K., Phelan N., Feeney J., Fitzgibbon M., Boran G., O’Mara S., Gibney J. (2009). Evidence for a specific defect in hippocampal memory in overt and subclinical hypothyroidism. J. Clin. Endocrinol. Metab..

[65] Burmeister L.A., Ganguli M., Dodge H.H., Toczek T., DeKosky S.T., Nebes R.D. (2001). Hypothyroidism and cognition: Preliminary evidence for a specific defect in memory. Thyroid.

[66] Davis J.D., Tremont G. (2007). Neuropsychiatric aspects of hypothyroidism and treatment reversibility. Minerva Endocrinol..

[67] Gulseren S., Gulseren L., Hekimsoy Z., Cetinay P., Ozen C., Tokatlioglu B. (2006). Depression, anxiety, health-related quality of life, and disability in patients with overt and subclinical thyroid dysfunction. Arch. Med. Res..

[68] Constant E.L., Adam S., Seron X., Bruyer R., Seghers A., Daumerie C. (2005). Anxiety and depression, attention, and executive functions in hypothyroidism. J. Int. Neuropsychol. Soc..

[69] Kuhlmann L., Joensson I.M., Froekjaer J.B., Krogh K., Farholt S. (2014). A descriptive study of colorectal function in adults with Prader-Willi Syndrome: High prevalence of constipation. BMC Gastroenterol..

[70] Persani L., Ferretti E., Borgato S., Faglia G., Beck-Peccoz P. (2000). Circulating thyrotropin bioactivity in sporadic central hypothyroidism. J. Clin. Endocrinol. Metab..

[71] Horimoto M., Nishikawa M., Ishihara T., Yoshikawa N., Yoshimura M., Inada M. (1995). Bioactivity of thyrotropin (TSH) in patients with central hypothyroidism: Comparison between in vivo 3,5,3′-triiodothyronine response to TSH and in vitro bioactivity of TSH. J. Clin. Endocrinol. Metab..

[72] Lania A., Persani L., Beck-Peccoz P. (2008). Central hypothyroidism. Pituitary.

[73] Ramschak-Schwarzer S., Radkohl W., Stiegler C., Dimai H.P., Leb G. (2000). Interaction between psychotropic drugs and thyroid hormone metabolism—An overview. Acta Med. Austriaca.

[74] Sauvage M.F., Marquet P., Rousseau A., Raby C., Buxeraud J., Lachatre G. (1998). Relationship between psychotropic drugs and thyroid function: A review. Toxicol. Appl. Pharmacol..

[75] Bonnot O., Cohen D., Thuilleaux D., Consoli A., Cabal S., Tauber M. (2016). Psychotropic treatments in Prader-Willi syndrome: A critical review of published literature. Eur. J. Pediatrics.

[76] Bou Khalil R., Richa S. (2011). Thyroid adverse effects of psychotropic drugs: A review. Clin. Neuropharmacol..

[77] van der Spek A.H., Fliers E., Boelen A. (2017). The classic pathways of thyroid hormone metabolism. Mol. Cell Endocrinol..

[78] Bianco A.C., Salvatore D., Gereben B., Berry M.J., Larsen P.R. (2002). Biochemistry, cellular and molecular biology, and physiological roles of the iodothyronine selenodeiodinases. Endocr. Rev..

[79] Mullur R., Liu Y.Y., Brent G.A. (2014). Thyroid hormone regulation of metabolism. Physiol. Rev..

[80] Rezvani I., DiGeorge A.M., Dowshen S.A., Bourdony C.J. (1981). Action of human growth hormone (hGH) on extrathyroidal conversion of thyroxine (T4) to triiodothyronine (T3) in children with hypopituitarism. Pediatric Res..

[81] Jorgensen J.O., Pedersen S.A., Laurberg P., Weeke J., Skakkebaek N.E., Christiansen J.S. (1989). Effects of growth hormone therapy on thyroid function of growth hormone-deficient adults with and without concomitant thyroxine-substituted central hypothyroidism. J. Clin. Endocrinol. Metab..

[82] Behan L.A., Monson J.P., Agha A. (2011). The interaction between growth hormone and the thyroid axis in hypopituitary patients. Clin. Endocrinol..

[83] Pirazzoli P., Cacciari E., Mandini M., Sganga T., Capelli M., Cicognani A., Gualandi S. (1992). Growth and thyroid function in children treated with growth hormone. J. Pediatrics.

[84] Portes E.S., Oliveira J.H., MacCagnan P., Abucham J. (2000). Changes in serum thyroid hormones levels and their mechanisms during long-term growth hormone (GH) replacement therapy in GH deficient children. Clin. Endocrinol..

[85] Jorgensen J.O., Ovesen P., Juul A., Hansen T.K., Skakkebaek N.E., Christiansen J.S. (1999). Impact of growth hormone administration on other hormonal axes. Horm. Res..

[86] Agha A., Walker D., Perry L., Drake W.M., Chew S.L., Jenkins P.J., Grossman A.B., Monson J.P. (2007). Unmasking of central hypothyroidism following growth hormone replacement in adult hypopituitary patients. Clin. Endocrinol..

[87] Smyczynska J., Hilczer M., Stawerska R., Lewinski A. (2010). Thyroid function in children with growth hormone (GH) deficiency during the initial phase of GH replacement therapy-clinical implications. Thyroid. Res..

[88] Butler M.G., Smith B.K., Lee J., Gibson C., Schmoll C., Moore W.V., Donnelly J.E. (2013). Effects of growth hormone treatment in adults with Prader-Willi syndrome. Growth Horm. IGF Res..

[89] Cettour-Rose P., Burger A.G., Meier C.A., Visser T.J., Rohner-Jeanrenaud F. (2002). Central stimulatory effect of leptin on T3 production is mediated by brown adipose tissue type II deiodinase. Am. J. Physiol. Endocrinol. Metab..

[90] Reinehr T. (2010). Obesity and thyroid function. Mol. Cell Endocrinol..

[91] Tauber M., Coupaye M., Diene G., Molinas C., Valette M., Beauloye V. (2019). Prader-Willi syndrome: A model for understanding the ghrelin system. J. Neuroendocrinol..

[92] Cummings D.E., Clement K., Purnell J.Q., Vaisse C., Foster K.E., Frayo R.S., Schwartz M.W., Basdevant A., Weigle D.S. (2002). Elevated plasma ghrelin levels in Prader Willi syndrome. Nat. Med..

[93] Kordi F., Khazali H. (2015). The effect of ghrelin and estradiol on mean concentration of thyroid hormones. Int. J. Endocrinol. Metab..

