# Supplementary material for: Thyroid Function in Adults with Prader–Willi Syndrome; a Cohort Study and Literature Review
Source: J Clin Med. 2021 Aug 25;10(17):3804. doi: 10.3390/jcm10173804 (PMC8432005; doi:10.3390/jcm10173804)
Supplement: Supplementary file 1 [file jcm-10-03804-s001.zip › jcm-1300429-supplementary.pdf]

Supplementary Materials

Table S1. Full search strategy (Embase).

('Prader Willi syndrome'/exp OR 'mkrn3 gene'/de OR 'makorin ring finger protein 3'/de OR 'magel2 gene'/de OR 'magel2 protein'/de OR 'necdin'/de OR 'small nuclear ribonucleoprotein polypeptide n'/de OR 'snrpn gene'/de OR 'snrpn protein'/de OR 'e6 associated protein'/de OR ((Prader* NEAR/3 Willi*) OR PraderWilli OR mkrn3 OR makorin-3 OR znf127 OR D15S9 OR RNF63 OR EC-2.3.2.27 OR ZFP127 OR CPPB2 OR magel2 OR magel-2 OR ((makorin) NEAR/3 (ring) NEAR/3 (3)) OR ((zinc) NEAR/3 (finger) NEAR/3 (127)) OR ((ring) NEAR/3 (finger) NEAR/3 (63)) OR ((MAGE OR melanoma) NEAR/3 (family) NEAR/3 (L2)) OR ((necdin-like OR MAGE-like) NEAR/3 (protein) NEAR/3 (1 OR 2)) OR NDNL1 OR MAGE-Like-2 OR SHFYNG OR PWLS OR NM15 OR (NDN NOT nonclassic-differentiation-number*) OR necdin* OR HsT16328 OR PWCR OR C15orf2 OR NPAP1 OR NPAP-1 OR Nuclear-Pore-Associated-Protein-1 OR ((chromosome-15) NEAR/3 (open-reading-frame* OR ORF*) NEAR/3 (2)) OR SNURF-SNRPN* OR SNRPN* OR Small-Nuclear-Ribonucleoprotein-Polypeptide-N* OR Small-Nuclear-Ribonucleoprotein-Associated-Protein-N* OR Tissue-Specific-Splicing-Protein* OR Sm-Protein-D OR SM-Protein-N OR HCERN3 OR (((Sm-N OR SMN OR Sm-D OR PET1 OR BEY OR PED) AND (gene OR genes OR genetic OR genome* OR genomic)) NOT (sec*-malign*-neoplas* OR survival-motor-neuro*)) OR Small-Nucl*-Ribonucleoprot*-N OR RT-LI OR PWCR OR SNORD107 OR SNORD-107 OR (("C/D" OR CD) NEAR/3 (box*) NEAR/3 (107 OR 64 OR 109A OR 116 OR 115 OR 109B)) OR HBII-436 OR RF01164 OR SNORD64 OR HBII-13 OR RF00570 OR SNORD109A OR HBII-438A OR RF01278 OR SNORD116 OR HBII-85 OR PWCR1 OR SNORD115 OR HBII-52 OR RNHBII52 OR RF00105 OR SNORD109B OR HBII-438B OR RF01278 OR KIAA1899):ab,ti,kw) AND ('thyromimetic agent'/exp OR 'thyrotropin derivative'/exp OR 'antithyroid agent'/exp OR 'protirelin derivative'/exp OR 'thyroid disease'/exp OR (thyro* OR hyperthyro* OR hypothyro* OR euthyro* OR thyreo* OR TSH OR triiodothyronine* OR liothyroni* OR antithyro* OR iodine* OR iodo* OR triiodi* OR diiodi* OR monoiodi* OR tetraiodi* OR deiodinase* OR protirelin* OR TRH):ab,ti,kw) NOT ([Conference Abstract]/lim AND [1800-2017]/py)

The search strategy was adjusted, but the same search terms were used for the databases Medline ALL, the Web of Science Core Collection, the Cochrane Central Register of Controlled Trials, and Google Scholar. Additionally, references were searched.

Table S2. Prevalence of hypothyroidism and thyroid hormone levels in relation to use of psychotropic drugs (Part 1).

	Missing	Total *n* = 122	Psychotropic Drugs *n* = 49	No Psychotropic Drugs *n* = 73	*p*-Value	Atypical Antipsychotics (Non Phenothiazine) *n* = 20	No Atypical Antipsychotics *n* = 102	*p*-Value
n of males, *n* of females	0	58, 64	25, 24	33, 40	NA	10, 10	48, 54	NA
Hypothyroidism, *n* (%)	0	21 (17%)	10 (20%)	11 (15%)	0.5	5 (25%)	16 (16%)	0.3
Subclinical hypothyroidism, *n* (%)	0	3 (2%)	1 (2%)	2 (3%)	NA	0 (0%)	3 (3%)	NA
Hyperthyroidism, *n* (%)	0	1 (1%)	0 (0%)	1 (1%)	NA	0 (0%)	1 (1%)	NA
n of males, *n* of females with normal thyroid function	0	52, 45	21, 17	31, 28	NA	8, 7	44, 38	NA
fT4 (pmol/L), median (IQR) (*n* = 97)	2	16.5 (14.3-18.5)	16.6 (14.7-18.7)	16.3 (14.1-18.1)	0.8	17.2 (14.6-18.7)	16.3 (14.3-18.2)	0.5
T3 (nmol/L), median (IQR) (*n* = 97)	52	1.9 (1.7-2.3)	1.7 (1.6-2.0)	2.1 (1.7-2.3)	0.02	2.1 (1.8-NA)	1.8 (1.7-2.3)	0.7
TSH (mU/L), median (IQR) (*n* = 97)	0	1.6 (1.1-2.3)	1.5 (0.9-2.5)	1.7 (1.2-2.2)	0.7	1.5 (0.9-2.3)	1.6 (1.2-2.3)	0.3

Abbreviations: not applicable (NA), free thyroxine (fT4), triiodothyronine (T3), thyroid stimulating hormone (TSH). Laboratory measurements are for patients with normal thyroid function only (*n* = 97).

Table S3. Prevalence of hypothyroidism and thyroid hormone levels in relation to use of psychotropic drugs (Part 2).

	Nontricyclic Antidepressants *n* = 10	No Nontricyclic Antidepressants *n* = 112	*p*-Value	Valproic Acid *n* = 12	No Valproic Acid *n* = 110	*p*-Value	Benzodiazepines *n* = 14	No Benzodiazepines *n* = 108	*p*-Value
n of males, *n* of females	5, 5	53, 59	NA	8, 4	50, 60	NA	8, 6	50, 58	NA
Hypothyroidism, *n* (%)	3 (30%)	18 (16%)	0.3	2 (17%)	19 (17%)	0.9	1 (7%)	20 (19%)	0.3
Subclinical hypothyroidism, *n* (%)	0 (0%)	3 (3%)	NA	0 (0%)	3 (3%)	NA	0 (0%)	3 (3%)	NA
Hyperthyroidism, *n* (%)	0 (0%)	1 (1%)	NA	0 (0%)	1 (1%)	NA	0 (0%)	1 (1%)	NA
n of males, *n* of females with normal thyroid function	5, 2	47, 43	NA	6, 4	46, 41	NA	7, 6	45, 39	NA
fT4 (pmol/L), median (IQR) (*n* = 97)	17.4 (16.5-20.0)	16.2 (14.3-18.2)	0.2	14.9 (14.2-17.8)	16.5 (14.5-18.6)	0.3	17.2 (16.2-19.4)	16.2 (14.3-18.1)	0.1
T3 (nmol/L), median (IQR) (*n* = 97)	1.7 (1.6-1.8)	2.0 (1.7-2.3)	0.1	2.1 (1.7-2.5)	1.9 (1.6-2.2)	0.3	1.7 (1.5-1.8)	2.0 (1.7-2.3)	0.1
TSH (mU/L), median (IQR) (*n* = 97)	1.8 (0.9-2.9)	1.6 (1.1-23)	0.8	2.1 (1.5-2.5)	1.6 (1.0-2.2)	0.1	1.3 (0.9-2.1)	1.7 (1.1-2.3)	0.2

Abbreviations: not applicable (NA), free thyroxine (fT4), triiodothyronine (T3), thyroid stimulating hormone (TSH). Laboratory measurements are for patients with normal thyroid function only (*n* = 97).

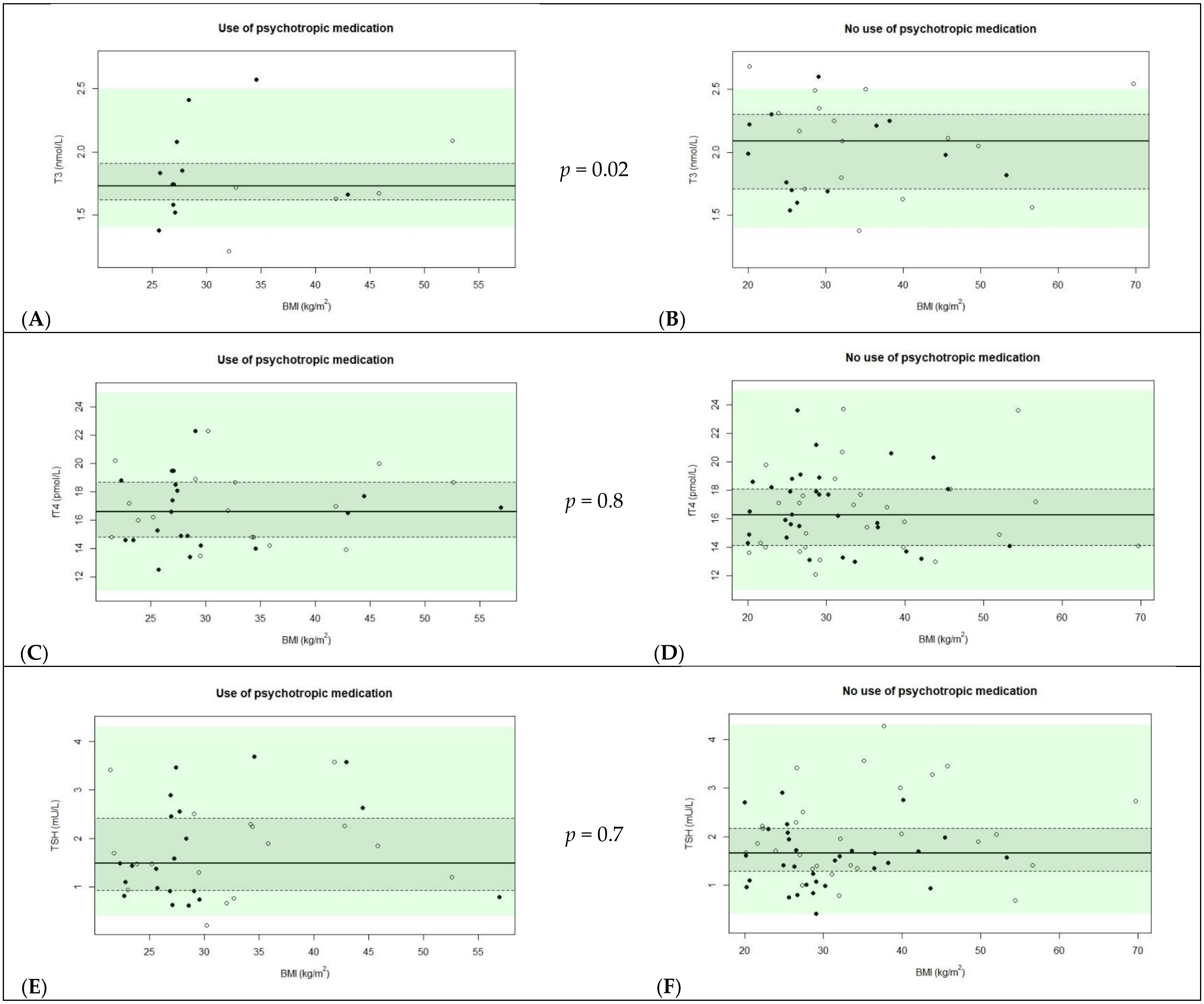

Figure S1. Scatterplot of T3, fT4, and TSH in relation to BMI for patients who use psychotropic drugs versus patients who do not.

Abbreviations: body mass index (BMI), free thyroxine (fT4), triiodothyronine (T3), thyroid stimulating hormone (TSH). Only patients without hyperthyroidism, hypothyroidism or subclinical hypothyroidism are depicted in this figure. *p*-values for the relationship between the use of psychotropic drugs and the thyroid hormone concentrations (T3, fT4 and TSH respectively) are shown in the middle.

Legends: males are depicted by closed dots and females by open dots. The solid line is the median and the dashed line the interquartile range. The reference range is given in as a green, transparent rectangle. (**A**) T3 vs. BMI for patients who use psychotropic drugs. (**B**) T3 vs. BMI for patients who do not use psychotropic drugs. (**C**) fT4 vs. BMI for patients who use psychotropic drugs. (**D**) fT4 vs. BMI for patients who do not use psychotropic drugs. (**E**) TSH vs. BMI for patients who use psychotropic drugs. (**F**) TSH vs. BMI for patients who do not use psychotropic drugs.

Reference values: TSH: before 1 February, 2019: 0.4-4.3 mU/L (n=69), after 1 February, 2019: 0.56-4.27 mU/L (n=28). fT4: before 12 April, 2019: 11-25 pmol/L (n=73), after 12 April, 2019: 13.5-24.3 pmol/L (n=22). T3: before 12 April, 2019: 1.4-2.5 (n=33), after 12 April, 2019: 0.7-2.0 nmol/L (n=12). Only the reference range that was valid for the most observations is shown.

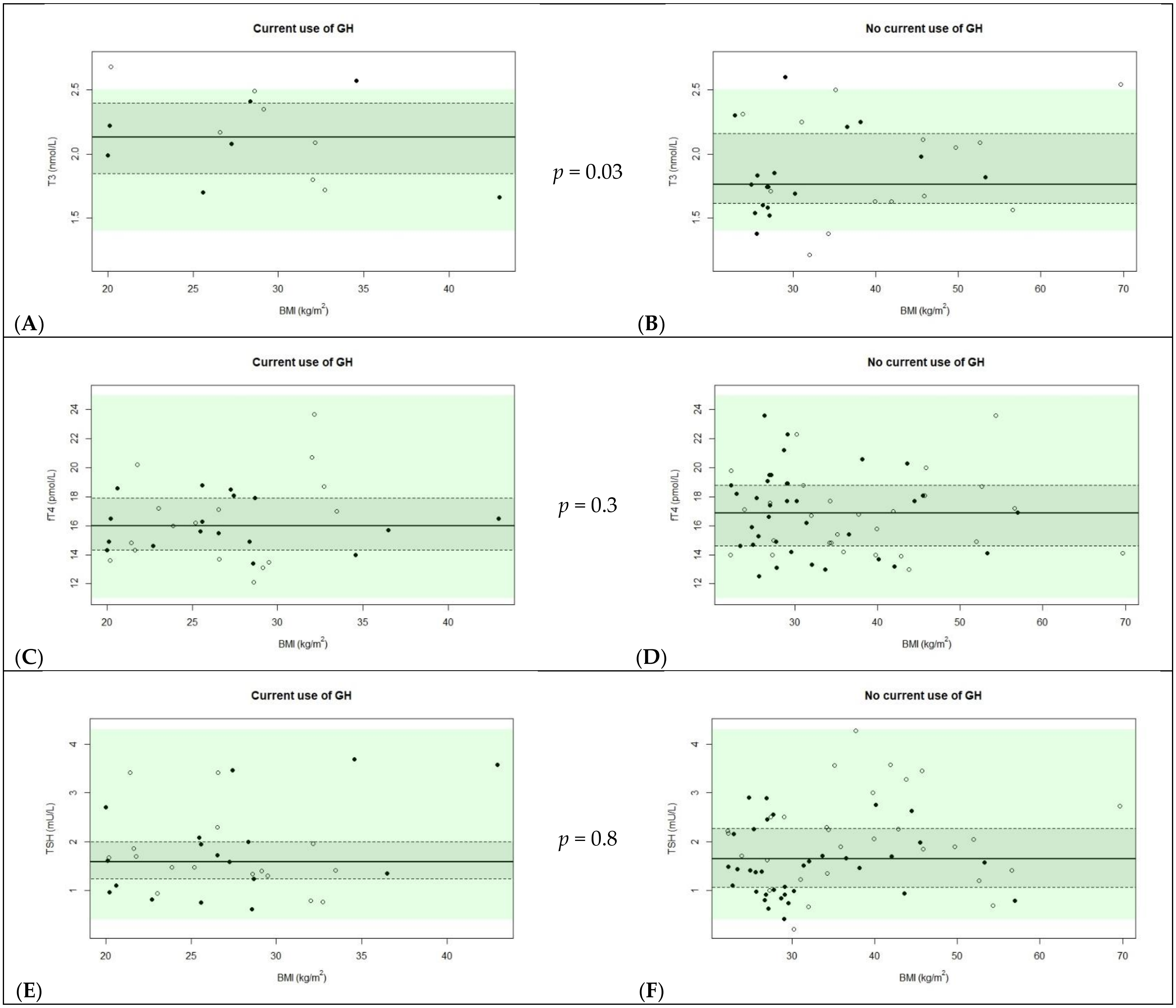

Figure S2. Scatterplot of T3, fT4, and TSH in relation to BMI for patients who currently use growth hormone treatment versus patients who do not.

Abbreviations: body mass index (BMI), free thyroxine (fT4), triiodothyronine (T3), thyroid stimulating hormone (TSH). Only patients without hyperthyroidism, hypothyroidism or subclinical hypothyroidism are depicted in this figure. *p*-values for the relationship between the current use of growth hormone treatment and the thyroid hormone concentrations (T3, fT4 and TSH respectively) are shown in the middle.

Legends: males are depicted by closed dots and females by open dots. The solid line is the median and the dashed line the interquartile range. The reference range is given in as a green, transparent rectangle. (**A**) T3 vs. BMI for patients who currently use growth hormone. (**B**) T3 vs. BMI for patients who do not currently use growth hormone. (**C**) fT4 vs. BMI for patients who currently use growth hormone. (**D**) fT4 vs. BMI for patients who do not currently use growth hormone. (**E**) TSH vs. BMI for patients who currently use growth hormone. (**F**) TSH vs. BMI for patients who do not currently use growth hormone.

Reference values: TSH: before 1 February, 2019: 0.4-4.3 mU/L (n=69), after 1 February, 2019: 0.56-4.27 mU/L (n=28). fT4: before 12 April, 2019: 11-25 pmol/L (n=73), after 12 April, 2019: 13.5-24.3 pmol/L (n=22). T3: before 12 April, 2019: 1.4-2.5 (n=33), after 12 April, 2019: 0.7-2.0 nmol/L (n=12). Only the reference range that was valid for the most observations is shown.
